# Cognitive Impairment in Marginally Housed Youth: Prevalence and Risk Factors

**DOI:** 10.3389/fpubh.2019.00270

**Published:** 2019-10-08

**Authors:** Kristina Waclawik, Andrea A. Jones, Skye P. Barbic, Kristina M. Gicas, Tiffany A. O'Connor, Geoffrey N. Smith, Olga Leonova, Steve Mathias, Alasdair M. Barr, Ric M. Procyshyn, Donna J. Lang, Melissa L. Woodward, G. William MacEwan, William J. Panenka, Aiko Yamamoto, William G. Honer, Allen E. Thornton

**Affiliations:** ^1^Department of Psychology, Simon Fraser University, Burnaby, BC, Canada; ^2^Department of Psychiatry, University of British Columbia, Vancouver, BC, Canada; ^3^Department of Occupational Science and Occupational Therapy, University of British Columbia, Vancouver, BC, Canada; ^4^Centre for Health Evaluation and Outcome Sciences, Vancouver, BC, Canada; ^5^Providence Health Care and Foundry, Vancouver, BC, Canada; ^6^St. Paul's Hospital, Vancouver, BC, Canada; ^7^Department of Anesthesiology, Pharmacology and Therapeutics, University of British Columbia, Vancouver, BC, Canada; ^8^Department of Radiology, University of British Columbia, Vancouver, BC, Canada

**Keywords:** cognition, youth, young adults, marginalization, homeless, premorbid IQ, neurological soft signs, substance use

## Abstract

**Objective:** Homeless and marginally housed youth are particularly vulnerable members of society, and are known to experience numerous health problems, including psychiatric illness, substance use, and viral infection. Despite the presence of these risk factors for cognitive compromise, there is limited research on the cognitive functioning of homeless and marginally housed youth. The present study examines the degree and pattern of cognitive impairment and associations with key risk factors in a sample of marginally housed young adults.

**Method:** Participants (*N* = 101) aged 20–29 years old were recruited from single-room occupancy hotels, and underwent cognitive, psychiatric, neurological, and serological assessments.

**Results:** Forty percent of participants were identified as mildly cognitively impaired across multiple domains, and 16% were moderately-severely impaired. Deficits in memory and attention were most prevalent, while impairments in inhibitory control/processing speed and cognitive flexibility were also present but tended to be less severe. Developmental and historical factors (premorbid intellectual functioning, neurological soft signs, earlier exposure to and longer duration of homelessness or marginal housing), as well as current health risks (stimulant dependence and hepatitis C exposure), were associated with cognitive impairment.

**Conclusions:** The strikingly high rate of cognitive impairment in marginally housed young adults represents a major public health concern and is likely to pose a significant barrier to treatment and rehabilitation. These results suggest that the pathway to cognitive impairment involves both developmental vulnerability and modifiable risk factors. This study highlights the need for early interventions that address cognitive impairment and risk factors in marginalized young people.

## Introduction

Homeless and marginally housed youth are one of the most vulnerable sectors of the population (1, 2). Youth, defined here as spanning the ages of teenage to young adulthood [i.e., 15–29 years; (3)], are at a unique developmental period in which key skills necessary for adult functioning are acquired (1, 4). Both outright homelessness (i.e., living on the streets or in a shelter) and marginal housing [i.e., residing in temporary, unstable, and substandard living conditions; (5)] place youth in a vulnerable position in which risk of numerous negative outcomes is increased (6). Homeless and marginally housed youth have increased rates of numerous health challenges such as psychiatric illness, viral infection, and substance use (6, 7), all of which are known to compromise cognition (8–12). Cognitive impairment is a major obstacle to health and quality of life as it can hinder treatment access and preclude its effectiveness (13–15), portend poorer psychosocial functioning [e.g., (4, 16)], and pose a barrier to exiting homelessness and poverty (13). However, in comparison to children and adults, there is limited research on cognitive functioning in homeless or marginally housed youth. Prior reports document that homeless or marginally housed youth suffer cognitive impairment (1, 4, 17, 18), but the extent of impairment has been investigated in only a limited number of settings and the profile of deficits remains unclear. Additionally, while research has identified that psychiatric illness is a risk for impairment (1), a more comprehensive account of risk factors is lacking. This is one of the first reports on the cognitive functioning and associated risk factors of marginally housed youth. Here we characterize rates of cognitive impairment across several core domains and explore relationships with key risk factors, including substance use, psychiatric illness, viral infection, neurological abnormalities, premorbid cognitive functioning, and marginal housing history. Given that this is an emerging literature, the purpose of the present study was to survey several potential contributors to cognitive impairment to identify areas of focus for future research, and thus we did not have specific hypotheses about the relative strength of various risk factors.

## Methods

### Participants

Participants were recruited as part of an ongoing, 10-year longitudinal study conducted in accord with the Declaration of Helsinki and approved by the research ethics boards of the University of British Columbia and Simon Fraser University. Written informed consent was obtained after explaining the nature of the procedures. A full description of study recruitment and methods has been provided elsewhere (6, 19). Briefly, participants were recruited from single-room occupancy (SRO) hotels in the impoverished Downtown East Side neighborhood of Vancouver, Canada, as well as from the local community courthouse. The study inclusion criteria allowed all persons living in marginal housing (i.e., in an SRO, shelter, or on the street) to participate. Due to the recruitment design, 95% of participants resided in an SRO hotel at the time of their assessment. There was no exclusion on the basis of any clinical criteria. The present study utilized a subset of the sample who were under 30 years old at the time of study recruitment (*N* = 101), consistent with relevant policy definitions of young adulthood [e.g., (3)].

### Procedures

Four cognitive domains were evaluated in neuropsychological assessment: (1) verbal memory [Hopkins Verbal Learning Test-Revised (HVLT-R), (20)], (2) inhibitory control/processing speed [Stroop Color-Word Test, (21)], (3) sustained attention [A-prime score from the Rapid Visual Information Processing subtest from the Cambridge Neuropsychological Test Automated Battery (CANTAB), (22)], and (4) cognitive flexibility [total errors adjusted score of the Intra-Dimensional Extra-Dimensional subtest of the CANTAB, (22)]. For the Stroop, a composite score was created by averaging standardized scores for the three conditions (word-reading, color-naming, and color-word reading), given large correlations between these three scores (*r* = 0.61 −0.75). Similarly, an average of the standardized scores for HVLT-R total recall and delayed recall was used, as these two scores correlated at *r* = 0.75. Estimated premorbid intellectual functioning (IQ) was obtained via the predicted IQ score from the Wechsler Test of Adult Reading [WTAR; (23)], which takes into account WTAR reading score and demographic variables (age, gender, and education).

Psychiatric diagnoses were determined by a psychiatrist through the Best Estimate Clinical Evaluation and Diagnosis, according to the *Diagnostic and Statistical Manual for Mental Disorders-TR, Fourth Edition* (24). In order to capture neurodevelopment abnormalities, neurological soft signs were assessed using the total score from the Cambridge Neurological Inventory (25). To provide a non-developmental contrast, extrapyramidal symptoms (EPS) were assessed with the Extrapyramidal Symptom Rating Scale (26) and the Barnes Akathisia Rating Scale [BARS; (27)]. Extrapyramidal symptoms are considered to be non-developmental in origin (i.e., medication induced) yet share features of neurological soft signs (e.g., movement abnormalities). Serology tested for presence of antibodies for HIV, herpes simplex, hepatitis B, and hepatitis C, as well as quantitative polymerase chain reaction (qPCR) for active hepatitis C infection. At study entry participants also completed a sociodemographic questionnaire including information on housing history.

#### Prevalence of Cognitive Impairment

Prevalence of cognitive impairment was examined for individual cognitive domains, as well as for an estimate of general cognitive impairment which incorporated information on deficits across multiple domains. First, participants were classified as being mildly (1 to < 2 standard deviations below the normative mean) or moderately-severely (2 standard deviations or more below the normative mean) impaired within each domain. Second, general cognitive impairment was defined using the classification system from the well-established literature on HIV-related cognitive impairment (28, 29). Mild cognitive impairment was defined as 1 standard deviation below the normative mean on at least two cognitive domains, and moderate-severe impairment as 2 standard deviations below the mean on at least two domains.

#### Risk Factors for Cognitive Impairment

Logistic regression was conducted using Statistical Package for the Social Sciences (SPSS, Version 25) to evaluate risk factors for impairment within cognitive domains. For this analysis, cognitive impairment was dichotomized (1.5 standard deviation or more below the normative mean) in order to better cluster those with definitive functional impairment [e.g., (30, 31)] and to adhere to relevant statistical guidelines (32). Health risk factors were selected based upon prior empirical support for their association with cognition as well as modeling requirements (i.e., meeting prevalence rates sufficient for statistical analyses). Risk factors included opioid dependence, stimulant dependence, cannabis dependence, primary psychotic disorder diagnosis (schizophrenia or schizoaffective disorder), primary mood disorder (bipolar disorder I or II, or major depressive disorder), hepatitis C exposure, herpes simplex, premorbid IQ, neurological soft signs, extrapyramidal symptoms, age at first homelessness or marginal housing, duration of homelessness and marginal housing and gender. To avoid confounding duration of homelessness/marginal housing with age, a proportion variable was created (years spent in homelessness or marginal housing divided by current age). Each risk factor was entered in a separate model to obtain an unadjusted odds ratio.

## Results

### Participant Characteristics

A detailed description of this sample and numerous health characteristics has been provided elsewhere (6), and relevant descriptive information will be summarized briefly here. The average age of the participants was 25.10 years (*SD* = 2.90, range: 20–29 years), and 75% were male. Sixty-six percent of the sample self-identified as being of European descent, 40% as Indigenous, 7% as African-Canadian, and 6% as another ethnicity. Participants had high rates of major mental illness and substance dependence. The most prevalent mental health and substance use disorders included schizophrenia and schizoaffective disorder (28%), mood disorder (bipolar or major depression; 24%) opioid dependence (32%), stimulant dependence (57%), and cannabis dependence (59%). Other psychiatric illnesses and dependence on other substances were less frequent; for example, 16% had history of ADHD, 1% had FASD, and 19% had alcohol dependence. Sixty-three percent had herpes simplex. Thirty percent were positive for hepatitis C antibody, and 34% of those had active hepatitis C infection. Exposure to other viral infections, which are prevalent in middle-aged or older adult marginally housed samples (19, 33), were less common in this youth sample (5% for hepatitis B and 2% for HIV).

Most participants (69%) had not completed high school, with the average educational attainment of the sample at a grade 10 level (*M* = 10.60 years, *SD* = 1.47, range: 7–15 years). The estimated premorbid IQ of the sample was in the average range (*M* = 100.78, *SD* = 7.80). Only 8% of the participants had current part-time employment, and none were employed full-time. Most participants received income through welfare (56%), disability assistance (33%), or both (10%). Ninety-five percent of participants were living in an SRO, 4% in a shelter or on the street, and 1% in supportive transitional housing. Eighty-eight percent had been homeless at some point in the past. The average age at first homelessness or residence in marginal housing was 19.40 years (*SD* = 2.86, range 10–28 years), and average total duration of homeless and/or marginal housing was 3.86 years (range 0.14–16.50 years).

Percentages of missing data for neurocognitive measures ranged from 5% (Stroop) to 17% (RVP), due to computer malfunctions for the CANTAB or some participants declining to complete certain tests. Participants with incomplete data did not differ on any predictor variables (*p* > 0.05).

### Cognitive Impairment Prevalence and Risk Factors

**Figure 1:**
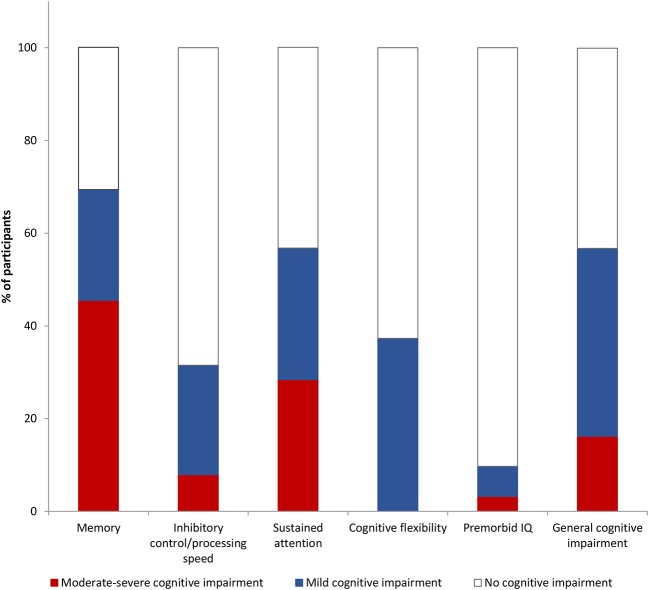
Rates of cognitive impairment in marginally housed youth.

Prevalence of mild and moderate-severe cognitive impairment is shown in Figure 1, and logistic regression results are shown in Table 1. Significant predictors for poorer memory included premorbid IQ, and duration of homelessness/marginal housing. Inhibitory control/processing speed impairment was associated with premorbid IQ. Cognitive flexibility impairment was associated with stimulant dependence and neurological soft signs. Sustained attention impairment was associated with premorbid IQ, age at first homelessness or marginal housing, duration of homelessness or marginal housing, and hepatitis C exposure. There were not enough participants with active hepatitis C infection (qPCR) to include this as a variable in the analysis (*n* = 8) and determine the extent to which active infection was driving the effect of hepatitis C exposure. However, in inspecting the means *post-hoc*, those with active hepatitis C infection had lower sustained attention scores (*M* = 31.55, *SD* = 11.60) than those who were hepatitis C antibody positive but without active infection (*M* = 41.06, *SD* = 22.62). Gender was also explored as a risk for memory and inhibitory control/processing speed impairments and was found to be non-contributory (memory OR = 0.66, *p* > 0.05, 95% CI: 0.25–1.76; inhibitory control/processing speed OR = 1.33, *p* > 0.05, 95% CI: 0.44–4.40). Note that the few females enrolled and the missing cognitive data rendered power inadequate for further gender analyses [see (32)].

**Table 1:** Associations between cognitive impairment and developmental and health risk factors.

**Descriptive information**	**Memory**	**Inhibitory control/processing speed**	**Sustained attention**	**Cognitive flexibility**
Percent at least 1.5 standard deviations below normative mean	52.00	23.50	37.70	36.60
*T* score (*M*, [*SD*])	34.30 (11.70)	44.04 (10.51)	38.44 (11.45)	42.33 (12.24)
**Unadjusted odds ratios (95% CI)**Reference group: unimpaired				
Premorbid IQ	0.91^**^ (0.86–0.97)	0.92^**^ (0.86–0.98)	0.93^*^ (0.86–0.99)	0.98 (0.92–1.04)
Neurological soft signs	1.13 (1.00–1.28)	1.07 (0.95–1.20)	0.98 (0.86–1.12)	1.16^*^ (1.01–1.32)
Extrapyramidal symptoms	1.81 (0.69–4.77)	1.33 (0.42–4.17)	1.12 (0.35–3.57)	2.39 (0.79–7.28)
Age at first homelessness/marginal housing	0.93 (0.84–1.05)	1.12 (0.98–1.27)	0.75^**^ (0.62–0.91)	1.12 (0.98–1.30)
Duration of homelessness/marginal housing (years)	1.05^*^ (1.01–1.09)	0.99 (0.95–1.04)	1.08^**^ (1.02–1.14)	1.01 (0.96–1.06)
Primary psychotic illness	1.40 (0.54–3.63)	0.66 (0.21–2.03)	0.43 (0.12–1.55)	1.32 (0.43–4.07)
Primary mood disorder	0.58 (0.21–1.57)	2.46 (0.84–7.18)	0.80 (0.23–2.79)	0.45 (0.13–1.59)
Stimulant dependence	2.01 (0.83–4.87)	1.39 (0.51–3.81)	1.65 (0.55–4.93)	3.56^*^ (1.16–10.89)
Opioid dependence	0.80 (0.28–2.25)	0.82 (0.28–2.41)	2.50 (0.82–7.60)	2.00 (0.70–5.34)
Cannabis dependence	1.12 (0.47–2.67)	0.83 (0.31–2.26)	2.13 (0.68–6.69)	1.34 (0.48–3.77)
Hepatitis C	0.79 (0.28–2.18)	0.32 (0.08–1.25)	4.00^*^ (1.14–14.00)	1.31 (0.40–4.34)
Herpes simplex	1.60 (0.58–4.46)	1.05 (0.35–3.15)	0.92 (0.27–3.16)	2.03 (0.61–6.72)

* *p < 0.05*,

** *p < 0.01*.

## Discussion

In a sample of marginally housed youth, a substantial proportion (over 40%) showed mild cognitive impairment (between 1 and 2 standard deviations below normative mean) across multiple domains, and an additional 16% were moderately-severely impaired (2 or more standard deviations below mean). Impairments were most prominent on memory and sustained attention tasks, with at least half of the youth classified as having impairment in these domains. In contrast, inhibitory control/processing speed and cognitive flexibility deficits were less common (in less than 40% of the sample), and tended to be less severe.

While past research in homeless or marginally housed children and adolescents has reported impairments in similar cognitive domains (1), our findings provide clarification as to the degree and pattern of impairment in young adults living in marginal housing. One of the few previous studies on cognition in marginally housed young adults also demonstrated high cognitive impairment, particularly for memory and to a lesser degree for attention, executive functions, and processing speed (4). However, the overall degree of impairment was lower in their sample, all of whom were participating in a supportive employment program with the majority currently employed (4). Our results indicate that cognitive impairment is more severe in a primarily unemployed sample, and indeed cognitive deficits likely pose a barrier to employment and other aspects of psychosocial functioning (4, 13).

We explored the relationship between cognitive impairment and several key risk factors that were present in this sample of marginally housed youth. Among a host of possible risk factors, several emerged as significant, including developmental, historical, and current health status factors. Of particular interest, the high rates of cognitive impairment we observed occur in the context of the sample's estimated premorbid IQ falling within normal limits. Yet, persons with lower estimated premorbid intellectual functioning were at greater risk of impairments across several cognitive domains. Greater neurological soft signs, an indicator of non-specific abnormal neurodevelopment (34), were also associated with cognitive impairment, as were younger age at first becoming homeless or marginally housed, and longer duration of homelessness or marginal housing. These findings suggest that individuals with pre-existing cognitive and neurological vulnerabilities may be particularly at risk for exhibiting significant impairment. Conversely, better premorbid functioning, likely reflecting cognitive reserve (35, 36), may protect against the effect of adverse environments and associated factors such as chronic stress, food insufficiency and malnutrition, and poor health (7, 37). In contrast to the aforementioned developmental and historical factors, most health risk factors in this sample did not predict cognitive impairment. However, the selective relationships with stimulant dependence and hepatitis C we observed may represent early effects of exposure, suggesting that certain health factors begin to play a role in compromising cognition early in adulthood. In middle-aged marginally housed samples, additional risk factors have also been linked to cognitive impairment, including HIV infection, psychiatric illness, and other substance dependence disorders (33, 38). The low prevalence of some risks (e.g., HIV) as well as shorter exposure duration in the youth is apt to account for divergence of the risk factors between the cohorts. Elucidating these differences is important as preventative interventions minimizing risk exposures of the youth may attenuate further cognitive decline.

In terms of limitations, the purpose of the present study was to conduct an initial survey of potential contributors to impairment that can be used as a starting point for future research in this emerging literature. Therefore, we treated each predictor independently and, consistent with our limited sample size, did not examine multivariate odds ratios. This is a limitation in the sense that we were not able to test for interaction effects between various risk factors, or control for potential third variables. Additionally, several health risks had prevalence rates so low as to prohibit analyses (i.e., HIV, alcohol dependence, and neurodevelopmental disorders such as ADHD and FASD). Given the low prevalence, these risks are unlikely to contribute substantially to the cognitive impairment of this particular population. Nonetheless, these risks may well impair cognition for the persons afflicted. Additionally, it is important to recognize the potential for a bidirectional relationship between risk factors and cognition. Poorer initial cognitive functioning could increase the likelihood of exposure to particular risk factors. For example, individuals with lower cognitive functioning may be at increased risk for becoming homeless at an earlier age due to poorer problem-solving skills, which could also contribute to reduced capacity for exiting homelessness. Future studies, particularly longitudinal studies, can help shed light on the interactive relationship between various risk factors and cognitive functioning in this complex population. Finally, we used a relatively brief cognitive battery and there were some cognitive and related domains that we did not assess (e.g., visuospatial memory, academic functioning). The battery was selected with the goal of targeting key cognitive domains using measures that are repeatable and engaging, and relatively brief in consideration of the testing length that could be tolerated in a generally low-functioning population. Future studies including comprehensive assessment of these and other cognitive domains will help further knowledge on the functioning of marginally housed youth.

The high prevalence of cognitive impairments observed here underscores the need for integrated interventions that take into account the significant cognitive barriers faced by this population, in addition to the numerous health risks. These findings highlight the need to prioritize treatment of the modifiable risk factors (such as hepatitis C exposure and stimulant dependence) in order to attenuate their impact on cognition. Other important interventions may include cognitive rehabilitation, which has shown promise in at-risk populations including homeless youth [e.g., (39–41)]. Such early interventions may help alter the trajectory of marginally housed youth and contribute to improved long-term prognosis.

In summary, the present work reveals endemic cognitive impairment in a cohort of marginally housed youth. These striking rates of cognitive impairment pose a substantial public health concern as they are apt to hinder employment, psychosocial functioning, and rehabilitation (4, 13). These findings further highlight the need for clinicians and integrated service delivery programs to address the significant cognitive challenges faced by marginalized young people.

## Data Availability Statement

The datasets generated for this study will not be made publicly available. Our individual level participant data includes identifiers of age, gender, employment, living situation, psychiatric diagnosis, and use of illicit drugs, all of which are needed for analyses. We cannot provide this data due to potential privacy infringement and related ethical and legal obligations to participants as restricted by the research ethics boards of the University of British Columbia and Simon Fraser University.

## Ethics Statement

This studies involving human participants were reviewed and approved by the Office of Research Ethics (University of British Columbia) and Research Ethics Board (Simon Fraser University). The patients/participants provided their written informed consent to participate in this study.

## Author Contributions

KW designed the research, analyzed and interpreted the data, and drafted the contribution. AJ, SB, and GS analyzed and interpreted the data, and made critical revision of the contribution for important intellectual content. KG and TO'C acquired the data and made critical revisions of the contribution for important intellectual content. OL acquired the data. RP, GM, and SM conceived and designed the research. DL and AY made critical revision of the contribution for important intellectual content. MW analyzed and interpreted the data. WP handled funding and supervision, and made critical revision to the contribution for important intellectual content. AB, WH, and AT conceived and designed the research, handled funding and supervision, and made critical revision of the contribution for important intellectual content. All authors contributed to manuscript revision, read and approved the submitted version.

### Conflict of Interest

RP has received speaking and Advisory Board fees from Janssen, Lundbeck, and Otsuka. He has also received Royalties as the Principal Editor of The Clinical Handbook of Psychotropic Drugs. GM has received speaking or consulting fees or sat on Advisory Boards for Apotex, AstraZeneca, Bristol-Myers Squibb, Janssen, Lundbeck, Otsuka, Pfizer, HLS, and Sunovion and has received research grant support from Janssen and Otsuka. WH has received consulting fees or sat on Advisory Boards for In Silico, Lundbeck, Otsuka, Alphasights, and Newron. AT has received grants from the William and Ada Isabelle Steel Fund and the Canadian Institutes of Health Research. The remaining authors declare that the research was conducted in the absence of any commercial or financial relationships that could be construed as a potential conflict of interest.
